# NEDD4 E3 ligase-catalyzed NAMPT ubiquitination and autophagy activation are essential for pyroptosis-independent NAMPT secretion in human monocytes

**DOI:** 10.1186/s12964-025-02164-5

**Published:** 2025-03-30

**Authors:** Marisela Rodriguez, Haifei Xu, Annie Hernandez, Julia Ingraham, Jason Canizales, Fernando Teran Arce, Sara M. Camp, Skyler Briggs, Aikseng Ooi, James M. Burke, Jin H. Song, Joe G.N. Garcia

**Affiliations:** 1 Center for Inflammation Science and Systems Medicine, University of Florida Scripps Research Institute, Jupiter, FL 33458 USA; 2 Department of Molecular Medicine, University of Florida Scripps Research Institute, Jupiter, FL USA

**Keywords:** eNAMPT, DAMP, Gasdermin D, Ubiquitination, NLRP3 inflammasome, Pyroptosis, Autophagy

## Abstract

NAMPT is an important intracellular metabolic enzyme (iNAMPT) regulating the NAD^+^ salvage pathway. However, increased cellular stress (infection, inflammation, hypoxia) promotes the secretion of extracellular NAMPT (eNAMPT), a TLR4 ligand and damage-associated molecular pattern protein (DAMP) that directly drives amplification of innate immune-mediated inflammatory, fibrotic, and neoplastic responses to influence disease severity. We sought to examine the mechanisms underlying pyroptotic eNAMPT release from human monocytic THP-1 cells, evoked by Nigericin, and non-pyroptotic eNAMPT secretion elicited by lipopolysaccharide (LPS). Our data indicate eNAMPT secretion/release requires NLRP3 inflammasome activation with substantial attenuation by either NLRP3 inhibition (MCC-950) or targeted genetic deletion of key inflammasome components, including NLRP3, caspase-1, or gasdermin D (GSDMD). Pyroptosis-associated eNAMPT release involved cleavage of the pore-forming GSDMD protein resulting in plasma membrane rupture (PMR) whereas non-pyroptotic LPS-induced eNAMPT secretion involved neither GSDMD cleavage nor PMR, verified utilizing non-cleavable GSDMD mutant constructs. LPS-induced eNAMPT secretion, however, was highly dependent upon NAMPT ubiquitination catalyzed by a complex containing the NEDD4 E3 ligase, Hsp90 (a selective chaperone), and intact GSDMD verified by enzymatic inhibition or silencing of NEDD4, GSDMD, or Hsp90. NAMPT ubiquitination and secretion involves autophagy activation as super-resolution microscopy analyses demonstrate NAMPT co-localization with autophagosome marker LC3B and eNAMPT secretion was significantly reduced by targeted ATG5 and ATG7 inhibition, critical components of the autophagy E3-like complex. These studies provide key insights into eNAMPT secretion that may accelerate the development of therapeutic strategies that address unmet therapeutic needs in inflammatory, fibrotic and neoplastic disorders.

**Supplementary Information:**

The online version contains supplementary material available at 10.1186/s12964-025-02164-5.

## Introduction

Nicotinamide phosphoribosyltransferase (NAMPT) is a homodimeric class II phosphoribosyltransferase, expressed in all mammalian tissues [1, 2], existing in two forms based upon intracellular (iNAMPT) or extracellular localization (eNAMPT) [3, 4]. iNAMPT is primarily located in the cytoplasm and nucleus [2] to function as the rate-limiting enzyme in the NAD^+^ biosynthetic salvage pathway [5] catalyzing the conversion of nicotinamide and 5′-phosphoribosyl-pyrophosphate (PRPP) into nicotinamide mononucleotide (NMN), a crucial NAD^+^ intermediate [6]. NMN is subsequently adenylated by NMNAT to produce NAD^+^, highlighting iNAMPT’s critical role in cellular metabolism and organismal survival [7, 8]. In contrast, eNAMPT is a secreted protein that was initially identified as a cytokine released by B lymphocytes to enhance pre-B-cell colony formation, (termed PBEF) [9], and has also been termed Visfatin due to adipokine functions upon release from visceral adipose tissue [10] via a process that appears to involve sirtuin 1 (SIRT1)-mediated iNAMPT deacetylation [11]. Overall, cell-specific mechanisms of eNAMPT secretion and release remain incompletely understood.

eNAMPT’s functional role as a critical innate immunity effector emanates from its participation as a damage-associated molecular pattern (DAMP) following cellular secretion or release triggered by increased stress, nutritional cues, or inflammatory signals. Unlike lipopolysaccharide (LPS), eNAMPT directly binds and activates the Toll-like receptor 4 (TLR4) without the requirement of cofactors or chaperones such as MD-2 (12) to promote inflammatory, fibrotic and neoplastic processes [12–14]. Elevated eNAMPT levels in the circulation correlate with the severity of acute and chronic inflammatory injuries [13, 15–17] and an eNAMPT-neutralizing antibody, ALT-100 mAb, currently in Phase 2A ARDS clinical trials (NCT05938036), has been shown to effectively reduce disease severity in preclinical models of acute respiratory distress syndrome (ARDS) [18–20], cardiac ischemia [21], pulmonary hypertension [22–24], lung and hepatic fibrosis [13, 25], colitis [26], lupus vasculitis [17] and various cancers [14, 27], Although the regulation of eNAMPT secretion/release under inflammatory, fibrotic and neoplastic conditions is poorly understood, multiple cellular stressors (infection, mechanical stress, ROS, hypoxia etc.) have been identified to induce *NAMPT* transcriptional activation driven by diverse transcription factors (SOX 17/18, HIF-2α, STAT5, NRF2, SP1, SREBP-1c) [23, 28, 29]. Discerning cell-specific mechanisms involved in eNAMPT secretion/release is fundamental to understanding eNAMPT’s involvement in human pathobiology.

In this regard, as NAMPT lacks an N-terminal secretion signal sequence required for the conventional pathway of protein secretion via endoplasmic reticulum (ER)-Golgi release in secretory vesicles or granules [9, 30], the current study explores the role of the unconventional protein secretion (UPS) pathways [31] in regulating eNAMPT secretion/release. This strategy is consistent with the observation that many UPS pathways, triggered by cellular stress, release proteins involved in signaling or immune responses. For example, current dogma defining mechanisms of interleukin-1β (IL-1β) secretion highlights a two-step activation model with the first signal (signal I) elicited by pathogen-associated molecular pattern proteins (PAMPs), such as the bacterial wall toxin, LPS, or DAMPs to induce pro-IL-1β expression as an inactive 32-kDa precursor. Effective IL-1β processing, maturation and secretion requires a second signal (signal II) under control of an inflammasome complex composed of a sensor protein (NLRP3), an adapter protein (ASC), and caspase-1 [32, 33] to convert pro-IL-1β to mature IL-1β [34, 35]. Nigericin, a potassium ionophore derived from the gram-negative bacterium *Streptomyces hygroscopicus*, elicits NLRP3 inflammasome and caspase-1 activation that results in gasdermin D (GSDMD) cleavage, releasing an effector N-terminal region (NT-GSDMD) [36] leading to the oligomerization of the NT-GSDMD fragments in the plasma membrane to form ring-shaped pores that lead to plasma membrane rupture (PMR), inflammatory cell death or pyroptosis [37] and release of mature IL-1β into the extracellular space [35, 36, 38–40].

Unlike the examination of IL-1β secretion, there is a paucity of studies interrogating mechanisms of eNAMPT secretion or release [10]. In this study, we utilized engineered human monocytic THP-1 cells to investigate the regulation of eNAMPT secretion/release by NLRP3 inflammasome activation and observed involvement of two distinct mechanisms, both dependent on NLRP3 inflammasome activation. eNAMPT release following pyroptotic induction is mediated by the pore-forming GSDMD with PMR whereas non-pyroptotic eNAMPT secretion elicited by LPS, requires an autophagosome-mediated secretory mechanism dependent upon eNAMPT ubiquitination catalyzed by the NEDD4 E3 ligase. We speculate that elucidating the key mechanisms driving eNAMPT secretion may allow for identification of novel therapeutic approaches to mitigate eNAMPT/TLR4 signaling and the severity of inflammatory, fibrotic and neoplastic injuries, a major unmet need.

## Materials and methods

### Cell lines and culture

THP-1 cells, including wild-type (cat# THP-null), NLRP3 knockout (NKO) (cat# THP-KO-NLRP3Z), GSDMD KO (cat# THP-KO-GSDMDZ), and caspase-1 knockdown (KD) (cat# THP-DCasp1), were purchased from InvivoGen. These cells were cultured in RPMI 1640 medium (Corning, Cat# 10-041-CV) supplemented with 10% fetal bovine serum (FBS; Gibco, cat# 26140-079) and 1% antibiotics (penicillin and streptomycin; Gibco, cat# 15140148). Cells were maintained at low passage numbers (≤ passage 20). HEK293T cells (ATCC, cat# CRL-11268) were cultured in Dulbecco’s modified Eagle’s medium (DMEM; Corning, cat# 10-013-CV)) with 10% FBS and 1% antibiotics. Both cell lines were grown in T75 flasks and incubated at 37 °C in a 5% CO2 atmosphere.

### Reagents and antibodies

Nigericin (cat# tlrl-nig), MCC-950 (cat# inh-mcc) and lipopolysaccharide (LPS; cat# tlrl-b5lps) were purchased from InvivoGen. Geldanamycin was from Fisher Scientific Gainesville, FL (Cat# 30562-34-6) and ATG7-IN-1 was from Targetmol Boston, MA (cat# T39705). Primary antibodies included NLRP3 (AdipoGen San Diego, CA, cat# 50-463-385), caspase-1 (AdipoGen, cat# 50-463-445), cleaved caspase-1 (Cell Signaling Technology, cat# 4199), Gasdermin D (Cell Signaling Technology, Cat# 39754), cleaved IL-1β (Cell Signaling Technology, cat# 83186), NAMPT (R&D Systems, cat# BAF4335; Bethyl Laboratories, cat# A300-778 A), HMGB1 (Abcam, cat# ab18256), Hsp90 (Cell Signaling Technology, cat# 4874; Santa Cruz Biotechnology, sc-13119), NEDD4 (R&D Systems, cat# MAB6218), Ubiquitin (Cell Signaling Technology, cat# 3936), ATG5 (Santa Cruz Biotechnology, cat# sc-133158), LC3B (Cell Signaling Technology, cat# 3868, and β-actin (Sigma-Aldrich, cat# A3854).

### Measurements of IL-1β, NAMPT, and HMGB1 levels in cell supernatants

Supernatants of THP-1 monocytes (5 × 10^6^ cells), retrieved after the indicated treatments, were centrifuged at 1,000 g for 5 min (4⁰C) and then used for the measurement of the levels of IL-1β and eNAMPT released or secreted into the culture medium using the Meso Scale Discovery ELISA platform as we previously reported [19, 20]. Separately, supernatants were also immediately concentrated using 10 kDa nominal molecular weight cutoff filters (Millipore-Sigma, cat# UFC501096) and then lysed in SDS-PAGE loading buffer. Western blot analysis was carried out for the detection of mature IL-1β (active), eNAMPT, HMGB1 in the cell-free supernatants along with detection of cleaved caspase-1 (active).

### Western blotting/immunoblotting and immunoprecipitation (IP)

Cell lysates in RIPA buffer (Thermo Scientific, cat# 89901) supplemented with protease and phosphatase inhibitors (Cell Signaling Technology, cat# 5872) were loaded in 4–12% protein gels (Bolt™ Bis-Tris Plus Mini Protein Gels) and transferred onto an Immuno-Blot polyvinylidene fluoride membranes (PVDF) membranes for western blotting studies as we previously described [41]. Immunoblots were visualized with Pierce chemiluminescent substrate (cat# 32106) for digital image acquisition using ChemiDoc MP Imager System (Bio-Rad). For co-immunoprecipitation, cleared lysates were subjected to IP by incubating with primary antibodies and agarose protein G Dynabeads (Invitrogen, cat #10003D) as indicated for 4–16 h at 4 °C. Following extensive washing with lysis buffer, bound proteins in Laemmli sample buffer were subjected to immunoblotting. For co-immunoprecipitation of transiently transfected HEK293 cells, cells were washed and lysed in RIPA buffer supplemented with protease and phosphatase inhibitors. Cleared lysates were subjected to IP by incubating with His-tag magnetic bead conjugate (Cell Signaling Technology, cat# 8811) at 4 °C.

### Plasmids, gene transduction and silencing

A shRNA NEDD4 set (ID: RHS4533-EG4734) including a pLKO.1 vector control (ID: RHS4080) were obtained from Horizon Discovery/Dhamacon and RHS3979-201739826 (Clone Id: TRCN0000007553) was selected for silencing NEDD4 expression. Mission shRNA pLKO.1-puro vector-ATG5 (TRCN0000151963), from Sigma-Aldrich were used to knockdown the expression of ATG5 in THP-1 cells. Lentiviruses were produced in Lenti-X HEK293 cells by Xfect (Takara Bio) based transfection of vector controls and the viral packaging plasmids psPAX2 and VSV-G (cat# 12260 and 12259; a gift from D. Trono, Addgene), followed by 0.45 µm filtration of virus-containing culture supernatants and concentration with Lent-X Concentrator (Takara, Cat# 631232). THP-1 cells were transduced with lentiviral particles in the presence of polybrene (8 µg ml^− 1^) and then subjected to spinoculation (800 g, 45 min, 32⁰C). Cells were puromycin selected (2 µg ml^− 1^) 72 hr after infection for 2 weeks. GSDMD Lentiviral Vector (Human) (CMV) (pLenti-GIII-CMV) (cat# 22730061) was obtained from Applied Biological Materials Inc. D275A point mutation was introduced by site-directed mutagenesis (Q5^®^ Site-Directed Mutagenesis Kit, NEB E0554S). The primer sequences are Fw: 5’-CTTCCTGACAgcgGGGGTCCCTG-3’, Rev 5’-TTGTGGAGGCACCTC-3’. HA-tagged human NEDD4 (cat# HG11437-NY) and Flag-tagged human GSDMD (cat# HG25207-NF) was ordered from Synobiological. His-tagged human NAMPT was described previously [41]. All constructs were verified by Sanger sequencing.

### Imaging studies

THP-1 cells were plated onto glass coverslips in 12-well plates (Fisher Scientific Co LLC: NC1418755) treated with PMA (Phorbol 12-myristate 13-acetate; Sigma: P1585-1MG) overnight to allow for cell adhesion to coverslip. Media was replaced with fresh media the following day and the cells were treated with respective treatments. After interval treatments, the cells were washed once with 1 mL of sterile RNAse free 1X PBS and fixed in 4% paraformaldehyde (PFA) for 15 min at room temperature. Washes of 1X PBS were completed (3Xs). A 15-minute incubation of 0.1% TritonX/PBS was used to permeabilize the cells followed by 3 washes of 1X PBS. Respective primary antibodies were treated and incubated overnight at 4⁰C. The following day, 1X PBS washes were repeated (3Xs) before secondary antibodies were applied and incubated for 2 h at 4⁰C. Wash with 1X PBS once and mount the coverslips onto slides with dual DAPI stain and fixing agent Vectashield (Vector Laboratories, 101098-044) and dried. Images were captured using a Nikon Eclipse Ti2 with a CFI60 Plan Apochromat Lambda D, a 100X oil immersion objective lens enabling better visualization of small target proteins with a numerical aperture of 1.45, W.D. 0.13 mm, F.O.V. 25 mm, DIC, Spring Loaded. A CSU-W1 Confocal Scanner Unit and Gataca live-SR super-resolution unit was used for super-resolution 3-dimensional imaging. Region of interest (ROI) co-localization of NAMPT fluorescence intensity within LC3B structures was performed utilizing ImageJ Analyze Particles tool after thresholding appropriately for LC3B puncta structures. Fluorescence intensity of cytoplasmic NAMPT foci was measured, excluding the nucleus.

### Live/Dead imaging and LDH release assay

Cell staining of live and dead was carried out using LIVE/DEAD^®^ Cell Imaging Kit, in which live cells are distinguished by conversion of non-fluorescent cell-permeant calcein AM to the intensely fluorescent calcein (FITC). In contrast, dying and dead cells are stained with cell-impermeant red probe that can only enter cells with damaged membranes binding to DNA (Texas Red). The staining image was taken with the EVOS M5000 Imaging System (Invitrogen). LDH activity was determined using the LDH Cytotoxicity Detection Kit (Thermo Fisher, cat# C20300) in freshly collected culture supernatants. In brief, 50 µL of supernatants placed in a 96 well flat-bottom plate were incubated with 50 µL of reaction mixture for 30 min in the dark at room temperature. Following incubation, 50 µL of supplied stop solution was added to each well, and the optical density (OD) values were determined by measurement of absorbance at 492 nm using a plate reading spectrophotometer. Additionally, the absorbance was read at 680 nm to account for any background absorbance values. Vehicle control in triplicate accounts for spontaneous LDH activity and 10X lysis buffer treatment accounts for a maximum LDH release. LDH percent release was calculated as follows for each absorbance: [(Compound treated LDH activity - spontaneous LDH activity/maximal LDH activity - spontaneous LDH activity)] x 100.

### Statistics and reproducibility

All representative results were independently repeated at least three times with similar results, and *n* indicates the number of biological replicates. Graphs were prepared in Prism 10 (GraphPad) and data are presented as mean values ± SEM. Numerical variables were analyzed using unpaired Student’s t-test or Bonferroni multiple comparison Test. Values of *P* < 0.05 were considered significant and marked by an asterisk.

## Results

### Nigericin promotes eNAMPT release via NLRP3 inflammasome activation and GSDMD-mediated plasma membrane rupture (PMR)

**Fig. 1:**
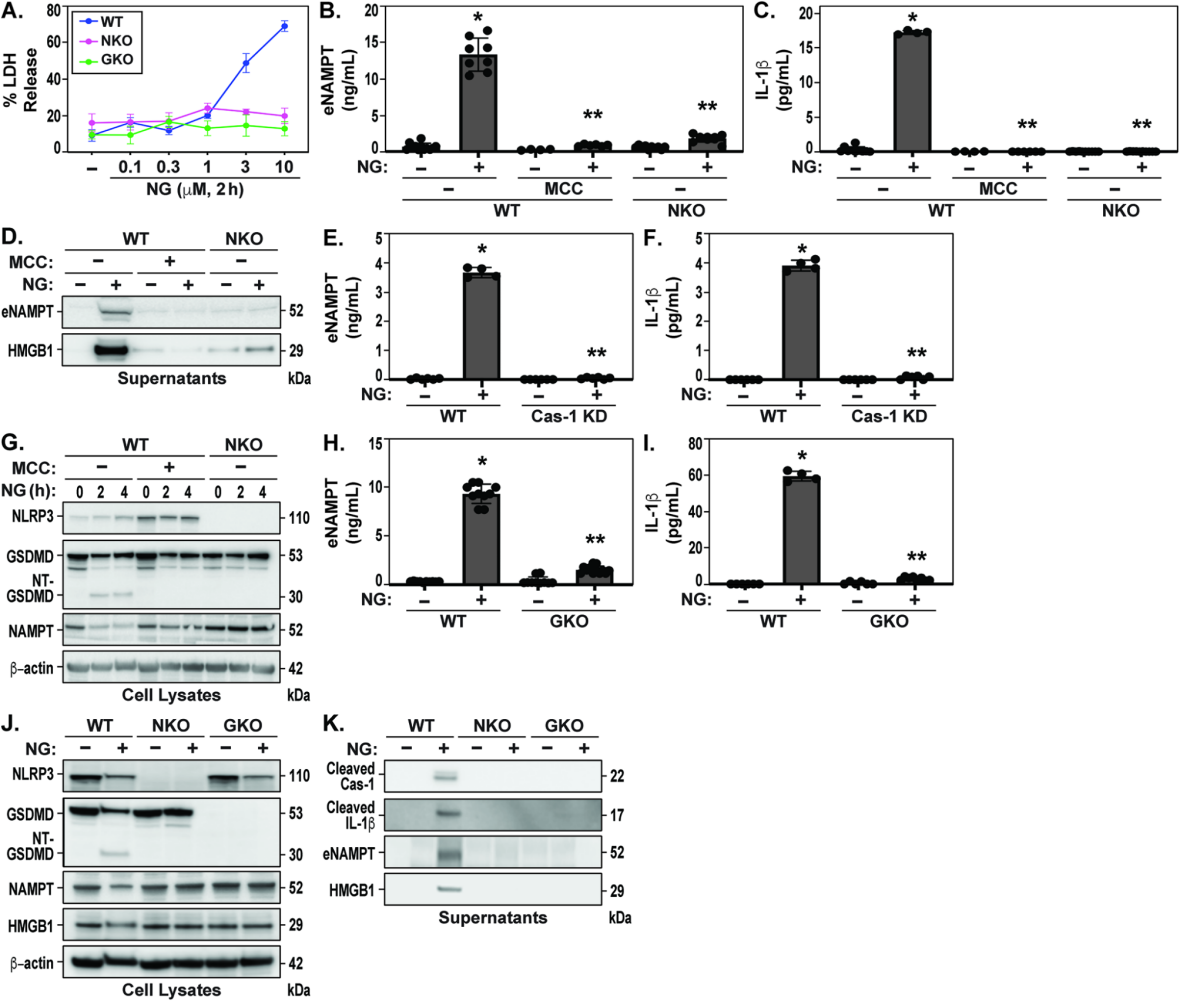
Nigericin promotes eNAMPT release via NLRP3 inflammasome activation and GSDMD-mediated plasma membrane rupture. (**A**) THP-1 wild type (WT), NLRP3 KO (NKO) and GSDMD KO (GKO) cells were challenged with Nigericin (0.1, 0.3, 1, 3 and 10 µM) (2 h). LDH release is depicted as mean +/- standard error mean (SEM) of three independent experiments. (**B**/**C**) eNAMPT and IL-1β release were measured in MCC-950 treated (1 h pretreatment) and untreated WT and NLRP3 KO (NKO) THP-1 cells by Meso Scale Discovery (MSD) assay 2 h after stimulation with 10 µM Nigericin. Data are shown as mean ± SEM of the means of four independent experiments. (**D**) Immunoblotting for supernatants of eNAMPT and HMGB1 release. (**E/F**) THP-1 WT and caspase-1 knockdown (Cas-1 KD) cells were challenged with 10 µM Nigericin treated (2 h). eNAMPT and IL-1β release was measured by MSD (mean +/- SEM of four independent experiments). (**G**) N-terminal GSDMD (NT-GSDMD) cleavage, NLRP3, NAMPT, and β-actin were analyzed by immunoblotting of cell lysates. MCC-950 pretreated (20 µM, 1 h) and untreated WT and NKO THP-1 cells were stimulated with Nigericin (10 µM, 2 and 4 h). (**H/I**) THP-1 WT and Gasdermin D (GSDMD) KO (GKO) THP-1 cells were challenged with Nigericin (10 µM, 2 h). eNAMPT and IL-1β releases were measured by MSD (mean +/- SEM of four independent experiments). (**J**) N-terminal GSDMD (NT-GSDMD) cleavage, NLRP3, NAMPT, HMGB1 and β-actin were analyzed by immunoblotting from cell lysates of Nigericin (10 µM, 2 h) treated and untreated THP-1 WT, NKO and GKO cells. (**K**) Supernatants of stimulated WT, NKO, and GKO THP-1 cells were immunoblotted for eNAMPT, HMGB1, cleaved IL-1β (active), and cleaved caspase-1 after the stimulation indicated. Statistical analysis t-test (B, C, E, F, H, I). Values are expressed as mean +/- SEM (*n* = 4, *p < 0.05 vs. WT, **p < 0.05 vs. WT + NG)

**Fig. 2:**
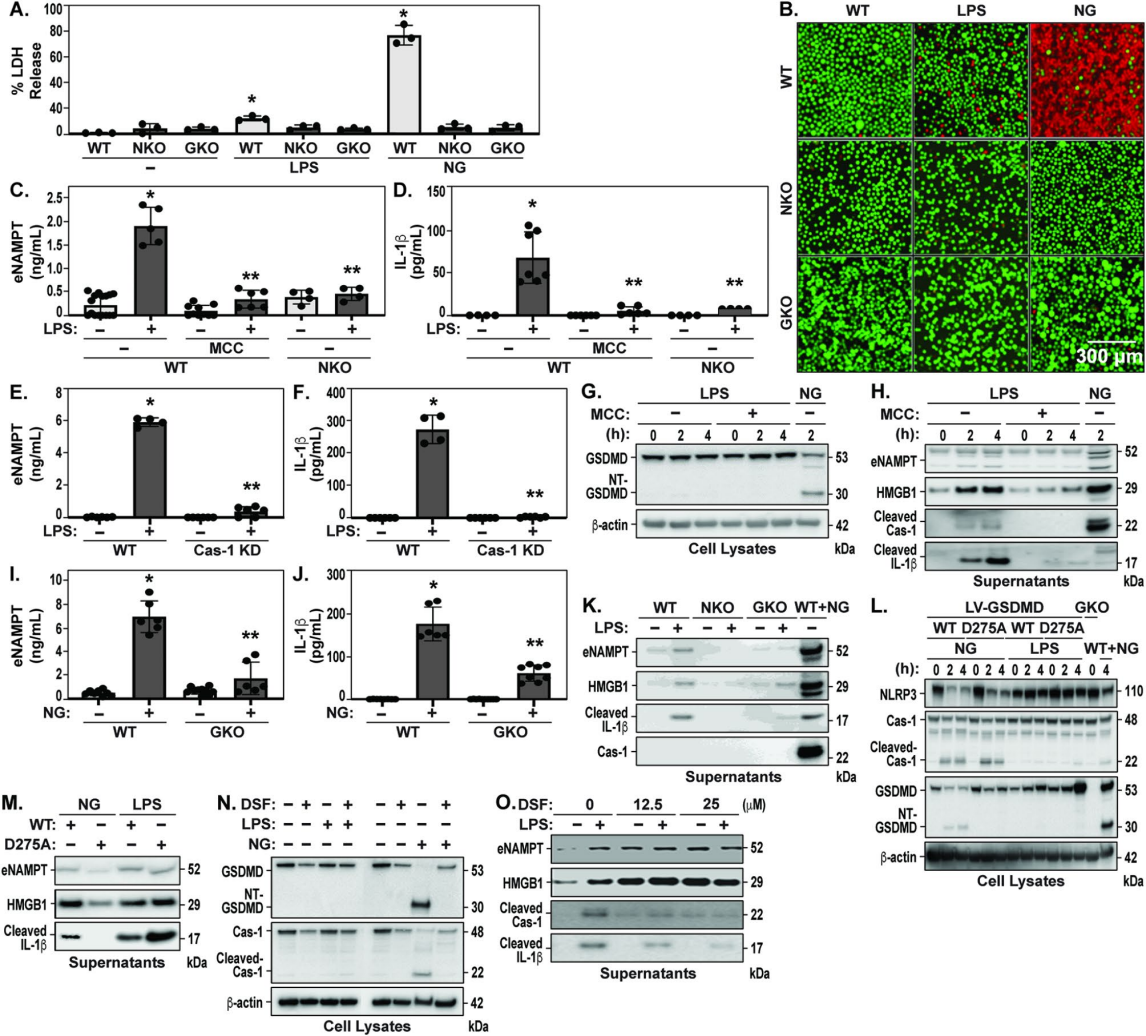
LPS induces GSDMD-dependent eNAMPT secretion via NLRP3 inflammasome activation without GSDMD cleavage or PMR. (**A**) THP-1 wild type (WT), NLRP3 KO (NKO) and GSDMD KO (GKO) cells were challenged with LPS (1 µg/mL, 4 h), or Nigericin (10 µM, 4 h). LDH release is depicted as mean +/- SEM of three independent experiments. (**B**) Cell membrane rupture is depicted by cell-impermeable red dye staining (dead) or permeable cell membrane integrity maintenance green dye staining. LIVE/DEAD viability/cytotoxicity between treatments indicated in (A) were captured using two-color dual parameter cell viability assay, imaged at 10X on EVOS microscope. (**C**/**D**) eNAMPT and IL-1β release were measured in MCC-950 pretreated (1 h) and untreated WT and NLRP3 KO (NKO) THP-1 cells by Meso Scale Discovery (MSD) assay 4 h after stimulation with 1 µg/mL LPS. Data are shown as mean ± SEM of four independent experiments. (**E/F**) THP-1 WT and caspase-1 knockdown (Cas-1 KD) cells were treated for 4 h with 1 µg/mL LPS. eNAMPT and IL-1β releases measured by MSD are depicted as mean +/- SEM of four independent experiments. (**G**) N-terminal GSDMD (NT-GSDMD) cleavage and β-actin were analyzed by immunoblotting from cell lysates. MCC-950 pretreated (20 µM, 1 h) and untreated WT THP-1 cells were treated for 2 and 4 h with 1 µg/mL LPS. 10 µM Nigericin (2 h); treated cells were used as comparison, which induced marked cleavages of NT-GSDMD. (**H**) Supernatants of THP-1 cells were immunoblotted against eNAMPT, HMGB1, cleaved IL-1β (active), and cleaved caspase-1 after the stimulation as indicated in (**G**). (**I/J**) THP-1 WT and gasdermin D KO (GKO) cells were treated for 4 h with 1 µg/mL LPS. eNAMPT and IL-1β release measured by MSD are depicted as mean +/- SEM of four independent experiments. (**K**) Supernatants of THP-1 WT, NKO and GKO cells were immunoblotted against eNAMPT, HMGB1, IL-1β (active) and cleaved caspase-1 after the stimulation of LPS (1 µg/mL, 4 h). Supernatants of 10 µM Nigericin (2 h) treated WT cells were used as a comparison. (**L**) WT GSDMD and D275A mutant GSDMD were reintroduced in GSDMD KO THP-1 cells using lentiviral transduction. These transduced cells were treated for 2 and 4 h with 10 µM Nigericin or 1 µg/mL LPS. Immunoblotting (IB) was performed to observe NLRP3, cleaved caspase-1 and NT-GSDMD cleavage. (**M**) Supernatants of GKO cells re-expressing WT GSDMD or D275A mutant GSDMD were immunoblotted against eNAMPT, HMGB1, and IL-1β (active) after the stimulation of Nigericin (NG; 10 µM, 2 h) and LPS (1 µg/mL, 4 h). (**N**) WT THP-1 cells were pretreated for 1 h with 25 µM DSF and then treated with 10 µM Nigericin (2 h) or 1 µg/mL LPS (4 h). Cell lysates were immunoblotted against GSDMD, NT-GSDMD, caspase-1, cleaved caspase-1, and β-actin (**O**) Supernatants of WT THP-1 cells were immunoblotted against eNAMPT, HMGB1, IL-1β (active), and cleaved caspase-1 after the stimulation of LPS (1 µg/mL, 4 h) in presence of 0, 12.5 and 25 µM DSF. Statistical analysis t-test (C, D, E, F, G, and H). Values are expressed as mean Standard error mean (*n* = 4, *p < 0.05 vs. WT, **p < 0.05 vs. WT + LPS)

To investigate whether eNAMPT secretion involves inflammasome activation, human monocytic THP-1 cells were challenged with the known NLRP3 inflammasome activator, Nigericin [34]. We utilized detection of lactate dehydrogenase (LDH) as an indicator of cell death and plasma membrane rupture elicited by Nigericin treatment in THP-1 cells. Nigericin-stimulated inflammasome activation in THP-1 cells resulted in detection of significant LDH release, indicative of PMR (Fig. 1A). Additionally, NLRP3 knockout (NKO) THP-1 cells or GSDMD knockout (GKO) THP-1 cells exhibited marked inhibition of Nigericin-induced LDH release and lytic cell death (Fig. 1A). Simultaneous staining of live cells with calcein AM and dead cells with ethidium homodimer-1 revealed increased red fluorescence of ethidium homodimer-1 (cell-impermeant viability indicator dye) in Nigericin-challenged THP-1 cells indicating significant loss of plasma membrane integrity consistent with PMR (as shown in Fig. 2B). Nigericin-challenged THP-1 cells with targeted deletion of NLRP3 or GSDMD exhibited preservation of plasma membrane integrity, results confirming that Nigericin activates the NLRP3 inflammasome leading to GSDMD-mediated pyroptosis and PMR.

We next observed that Nigericin-induced NLRP3 inflammasome activation and GSDMD cleavage resulted in robust increases of eNAMPT levels in supernatants from WT THP-1 cells (Fig. 1B). These responses were significantly attenuated by pretreatment with the NLRP3 inflammasome inhibitor, MCC-950, and abolished in Nigericin-challenged NKO cells (Fig. 1B). Nigericin-induced IL-1β release was similarly significantly reduced by MCC-950 pretreatment of WT THP-1 cells and in NKO cells (Fig. 1C), findings consistent with the requirement for NLRP3 inflammasome activation. Nigericin-induced NLRP3 inflammasome activation also resulted in the release of HMGB1, a key innate immunity DAMP [42, 43], into WT THP-1 cell supernatants (detected by Western blot analysis), with these elevations reduced in MCC-950 pretreated THP-1 WT cells and in Nigericin-challenged NKO cells (Fig. 1D).

As NLRP3 inflammasome activation is well recognized to involve caspase-1 cleavage/ activation, we next examined Nigericin-induced eNAMPT release in caspase-1-deficient THP-1 cells (Cas-1 KD) in comparison to WT THP-1 cells. Both eNAMPT (Fig. 1E) and IL-1β release (Fig. 1F) were significantly attenuated in Cas-1 KD THP-1 cells, supporting the requirement for NLRP3 oligomerization and subsequent caspase-1 activity for eNAMPT release [44]. Validating the role for caspase-1-mediated GSDMD cleavage in eliciting pyroptosis, we detected the 30 kDa NT-GSDMD cleaved fragment as well as NLRP3 inflammasome activation and caspase-1 activity in Nigericin-challenged THP-1 cells (Fig. 1G & Supplemental Fig. 1). These events occurred in association with rapid reductions in iNAMPT levels consistent with iNAMPT extracellular release via formation of NT-GSDMD pores or PMR. GSDMD pore formation is essential to both eNAMPT and IL-1β release as Nigericin-induced eNAMPT and IL-1β release was significantly reduced in GKO THP-1 cells (Fig. 1H/I). Similar to iNAMPT, intracellular HMGB1 levels were decreased concurrently with Nigericin-mediated GSDMD cleavage (NT-GSDMD) (Fig. 1J), a finding not observed in NLRP3 KO or GSDMD KO cells. This finding is consistent with increased levels of cleaved caspase-1, IL-1β, iNAMPT, and HMGB1 in supernatants from Nigericin-challenged WT THP-1 cells, abrogated in NKO or GKO THP-1 cells (Fig. 1K). Together, these data highlight the integral role of the NLRP3 inflammasome in pyroptotic release of DAMPs (eNAMPT and HMGB1), and cytokines (IL-1β) via sequential NLRP3 inflammasome activation, caspase-1 activation, GSDMD cleavage and pore formation, and PMR.

### LPS induces GSDMD-dependent eNAMPT secretion via NLRP3 inflammasome activation in the absence of GSDMD cleavage or PMR

LPS is a key PAMP and TLR4 ligand that primes the NLRP3 inflammasome in murine macrophages by promoting the transcriptional upregulation of inflammasome components through TLR4 activation [45]. In contrast, human monocytes do not require NLRP3 inflammasome priming to induce inflammasome activation [33, 46]. To interrogate the involvement of the NLRP3 inflammasome in LPS/TLR4 signaling- stimulated eNAMPT secretion and lytic cell death, THP-1 cells were challenged with LPS and cell viability assays performed measuring LDH release and fluorescence imaging of LIVE/DEAD cell assay for visualization of plasma membrane integrity. Unlike Nigericin, LPS challenge produced minimal effects on LDH release and alterations in plasma membrane integrity/pyroptosis in either WT THP-1 cells, NKO or GKO THP-1 cells (Figs. 2A/B). Measurements of eNAMPT levels in cell-free supernatants from LPS-challenged THP-1 cells showed significant increases although not achieving eNAMPT elevations comparable to Nigericin-challenged THP-1 cells. Both LPS-stimulated eNAMPT and IL-1β secretion were substantially inhibited by the NLRP3 inhibitor, MCC-950, and reduced in NKO THP-1 cells (Fig. 2C/D). Assessment of LPS-induced eNAMPT and IL-1β secretion in Cas-1 KD THP-1 cells showed near complete inhibition, confirming the essential involvement of NLRP3 inflammasome activation (Fig. 2E/F). Western blot analysis of eNAMPT and HMGB1 secretion in cell supernatants further validated the requirement for caspase-1 activation (Supplemental Fig. 2). These results indicate that LPS activates the NLRP3 inflammasome to facilitate the secretion of critical innate immunity DAMP and cytokine components such as eNAMPT, HMGB1, and IL-1β.

To investigate whether LPS-induced NLRP3 inflammasome activation results in GSDMD cleavage and NT-GSDMD oligomerization, Western blot analyses were performed on THP-1 cell lysates to detect immunoreactive, cleaved caspase-1 and GSDMD. Whereas Nigericin challenge produced marked pro-caspase-1 cleavage and production of the NT-GSDMD fragments, LPS induced increased detectable levels of cleaved caspase-1, cleaved IL-1β, eNAMPT and HMGB1 but without evidence of GSDMD cleavage in LPS-treated THP-1 cells (Fig. 2G). LPS-induced eNAMPT, IL-1β, and HMGB1 secretion was attenuated by MCC-950 pretreatment (Fig. 2H) with eNAMPT and IL-1β reduced in GKO THP-1 cells (Fig. 2I and J), results confirmed by Western blot analyses of supernatants including HMGB1, cleaved caspase-1, cleaved IL-1β (Fig. 2K).

To address the possibility that LPS-challenged THP-1 cells may facilitate eNAMPT, HMGB1, and IL-1β secretion via pore formation with GSDMD cleavage levels below detection limits, we re-expressed wild-type GSDMD (LV-GSDMD WT) and an uncleavable GSDMD mutant (LV-GSDMD D275A) in GKO THP-1 cells using lentiviral gene transduction. As expected, Nigericin-induced caspase-1 cleavage was comparable in both LV-GSDMD WT and LV-GSDMD D275A mutant cells, whereas GSDMD cleavage was detected only in LV-GSDMD WT cells (Fig. 2L). Neither WT-GSDMD nor D275A-GSDMD mutant cells exhibited GSDMD cleavage in response to LPS challenge (Fig. 2L). Western blot analysis of WT-GSDMD and D275A-GSDMD mutant cell supernatants showed that Nigericin-induced eNAMPT, HMGB1, and IL-1β secretion were significantly reduced or abrogated in LV-GSDMD D275A mutant cells (Fig. 2M) whereas eNAMPT, HMGB1, and IL-1β secretion was unaffected in LPS-challenged LV-GSDMD D275A mutant cells.

To further exclude the possible contribution of GSDMD pore formation to LPS-induced eNAMPT secretion, THP-1 cells were pretreated with disulfiram, a GSDMD oligomerization inhibitor [47], which resulted in inhibition of Nigericin-mediated caspase-1 NT-GSDMD cleavage (Fig. 2N). Disulfiram challenge did not alter LPS-induced eNAMPT and HMGB1 secretion (Fig. 2O) but significantly reduced IL-1β secretion presumably correlating with reduced levels of caspase-1 cleavage necessary for IL-1β maturation. Together, these data demonstrate that PMR and pyroptotic stimuli trigger conventional NLRP3 inflammasome activation to increase the release of eNAMPT, IL-1β, and HMGB1. In contrast, the non-pyroptotic stimulus, LPS, triggers non-canonical NLRP3 inflammasome activation to release eNAMPT, IL-1β, and HMGB1 in a manner clearly independent of pyroptosis and subsequent PMR.

### NEDD4 E3 ligase-mediated NAMPT ubiquitination is required for LPS-mediated NAMPT secretion: involvement of intact GSDMD and Hsp90

**Fig. 3:**
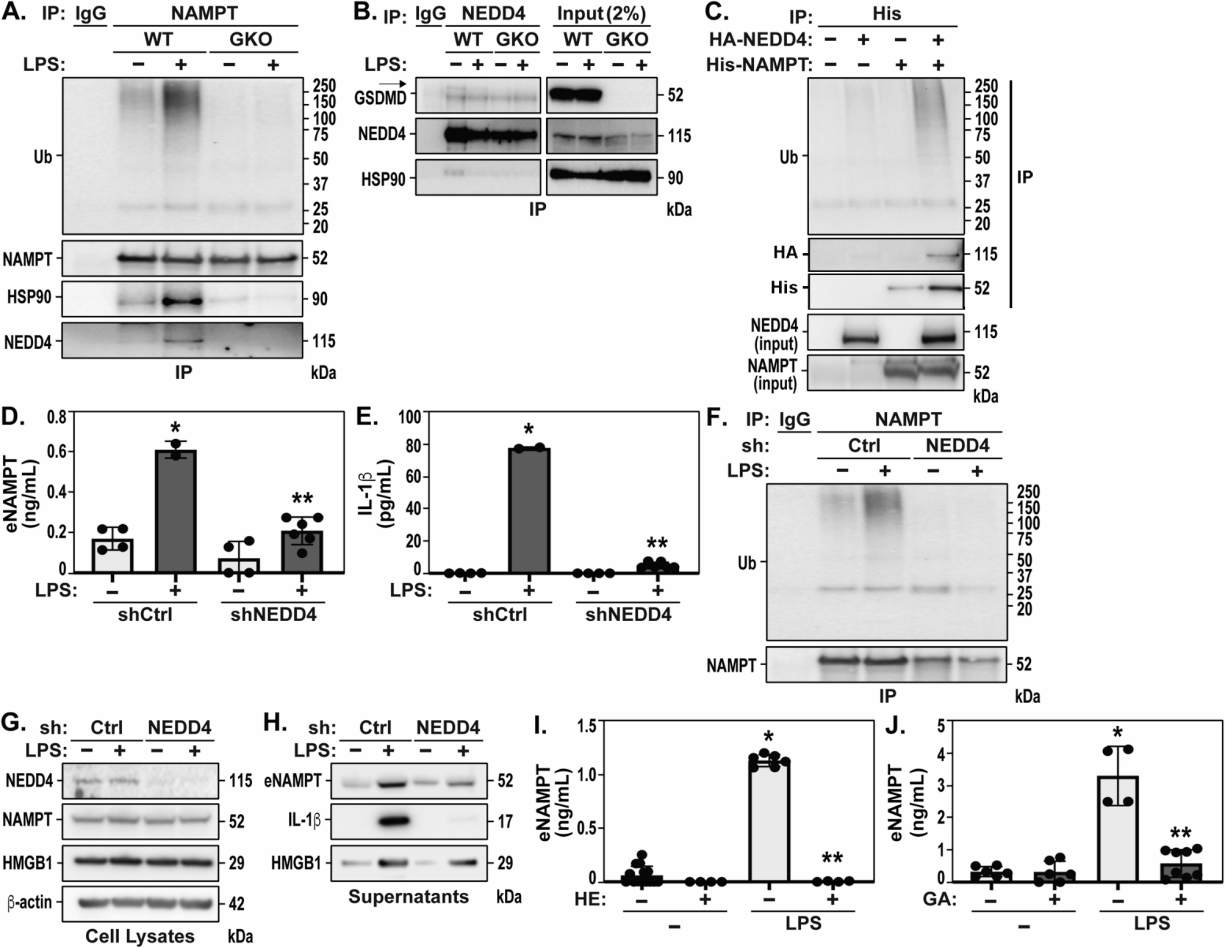
NEDD4 E3 ligase-mediated NAMPT ubiquitination is required for LPS-mediated NAMPT secretion: involvement of intact GSDMD and Hsp90. (**A**) NAMPT was immunoprecipitated with NAMPT antibody, and ubiquitination levels of NAMPT detected using anti-Ub antibodies after 4 h treatment of WT and GSDMD KO (GKO) THP-1 cells with 1 µg/mL LPS. NAMPT immunoprecipitants were immunoblotted (IB) with the indicated antibodies. NEDD4 and Hsp90 were coimmunoprecipitated with NAMPT in WT cells treated with LPS, but not in GKO cells. (**B**) NEDD4 was coimmunoprecipitated with GSDMD and Hsp90. A small portion (2% of cell lysates used for IP) of the total cell lysate was run on an IB alongside the immunoprecipitation (IP) samples to evaluate the efficiency of the IP. The arrow indicates the GSDMD band above the nonspecific bands. (**C**) HEK293T cells were co-transfected with the indicated plasmids for 24 h, and IP and IB were performed to observe NAMPT ubiquitination. (**D**/**E**) THP-1 cells expressing shNEDD4 and shControl were treated for 4 h with 1 µg/mL LPS. eNAMPT and IL-1β release measured by MSD are depicted as mean +/- SEM of four independent experiments. (**F**) shControl (shCtrl) and shNEDD4 transduced THP-1 cells were treated for 4 h with 1 µg/mL LPS. Cell lysates were immunoprecipitated with NAMPT antibody and IB were performed to observe NAMPT ubiquitination. (**G**) NEDD4 knockdown was achieved by lentiviral transduction of pLKO-shNEDD4 into THP-1 cells. IB detected protein expression levels of NEDD4, NAMPT, HMGB1, and β-actin in whole cell lysates (WCL) of THP-1 cells expressing shNEDD4 and shControl treated for 4 h with 1 µg/mL LPS. (**H**) Supernatants of shCtrl and shNEDD4 transduced THP-1 cells were immunoblotted against eNAMPT, HMGB1, and cleaved IL-1β (active) after the stimulation as indicated in (**G**). (**I**/**J**) THP-1 WT cells were pre-treated for 1 h with either 50 µM Heclin or 30 µM Geldanamycin (GA) and then treated for 4 h with 1 µg/mL LPS. eNAMPT release measured by MSD are depicted as mean +/- SEM of four independent experiments. Statistical analysis t-test. Values are expressed as mean Standard error mean (*n* = 4, D&E, *p < 0.05 vs. siCtrl, **p < 0.05 vs. LPS; I&E, *p < 0.05 vs. Ctrl, **p < 0.05 vs. LPS)

Recent studies have demonstrated that intact GSDMD plays a role in the non-pyroptotic release of IL-1β from LPS-exposed intestinal epithelial cells [48, 49]. This process is facilitated by the polyubiquitination of IL-1β through GSDMD-interacting proteins, including the E3 ligase NEDD4 and the chaperone Hsp90 (56, 58). We investigated whether LPS-mediated NAMPT secretion involves similar ubiquitination following NLRP3 inflammasome activation by LPS, involving intact GSDMD. Western blot studies of immunoprecipitated (IP) NAMPT from LPS-challenged WT THP-1 cells revealed significant NAMPT ubiquitination in a GSDMD-dependent manner as the interaction was abolished in GKO cells (Fig. 3A). Reciprocal analysis of NEDD4 IPs from THP-1 cells revealed the presence of a NEDD4-GSDMD-Hsp90 complex with the interaction of NEDD4 and Hsp90 abolished in THP-1 GKO cells and significant reduction of detectable NEDD4 protein expression levels (Fig. 3B). LPS stimulation appears to induce dissociation of Hsp90 from NEDD4 in WT THP-1 cells suggesting that in the presence of LPS, GSDMD and NEDD4 come together and Hsp90 facilitates the recruitment of NAMPT to the complex resulting in its ubiquitination. The interaction between NAMPT and NEDD4 was further investigated in co-IP experiments using His-tagged beads in HEK293T cells transfected with His-tagged NAMPT and hemagglutinin (HA)-tagged NEDD4 which led to direct binding of NEDD4 to NAMPT and enhanced NAMPT polyubiquitination in HEK293T co-transfectants expressing HA-NEDD4 and His-NAMPT (Fig. 3C).

We next examined whether NEDD4-mediated NAMPT ubiquitination promotes LPS-induced NAMPT secretion by silencing NEDD4 using lentiviral transduction of shRNA targeting NEDD4 in THP-1 cells (shNEDD4). This resulted in significantly reduced LPS-induced eNAMPT (Fig. 3D) and IL-1β (Fig. 3E) secretion, relative to WT cells. Importantly, NEDD4 silencing led to a significant reduction in NAMPT ubiquitination in response to LPS (Fig. 3F), confirming that NEDD4-mediated NAMPT ubiquitination is critical for eNAMPT secretion. NEDD4 silencing was confirmed via WB analysis of shNEDD4 THP-1 cell lysates (Fig. 3G). In contrast, the secretion of HMGB1 in the supernatants of shNEDD4 cells treated with LPS was not significantly altered when compared to shControl cells (Fig. 3H) suggesting that HMGB1 is not a substrate for NEDD4 ubiquitination and highlights the specificity of NEDD4’s role in NAMPT and IL-1β secretion. Pharmacological inhibition studies utilizing Heclin, an antagonist of the NEDD4 HECT domain, nearly abolished eNAMPT secretion (Fig. 3I) and reduced IL-1β levels (Supplemental Fig. 3) in LPS-challenged WT THP-1 cells, findings consistent with NEDD4 silencing. Finally, pharmacological inhibition studies were also employed to examine the crucial role of the Hsp90 chaperone protein in NAMPT secretion utilizing geldanamycin, a Hsp90 inhibitor, which effectively diminished eNAMPT secretion following LPS stimulation (Fig. 3J). Collectively, our results demonstrate that NLRP3 inflammasome activation by LPS leads to NAMPT secretion through a GSDMD-mediated pathway that promotes NAMPT ubiquitination via the E3 ubiquitin ligase NEDD4 HECT-specific domain.

### Ubiquitinated NAMPT is recruited to LC3B-containing autophagosomes facilitating autophagy-mediated secretion

**Fig. 4:**
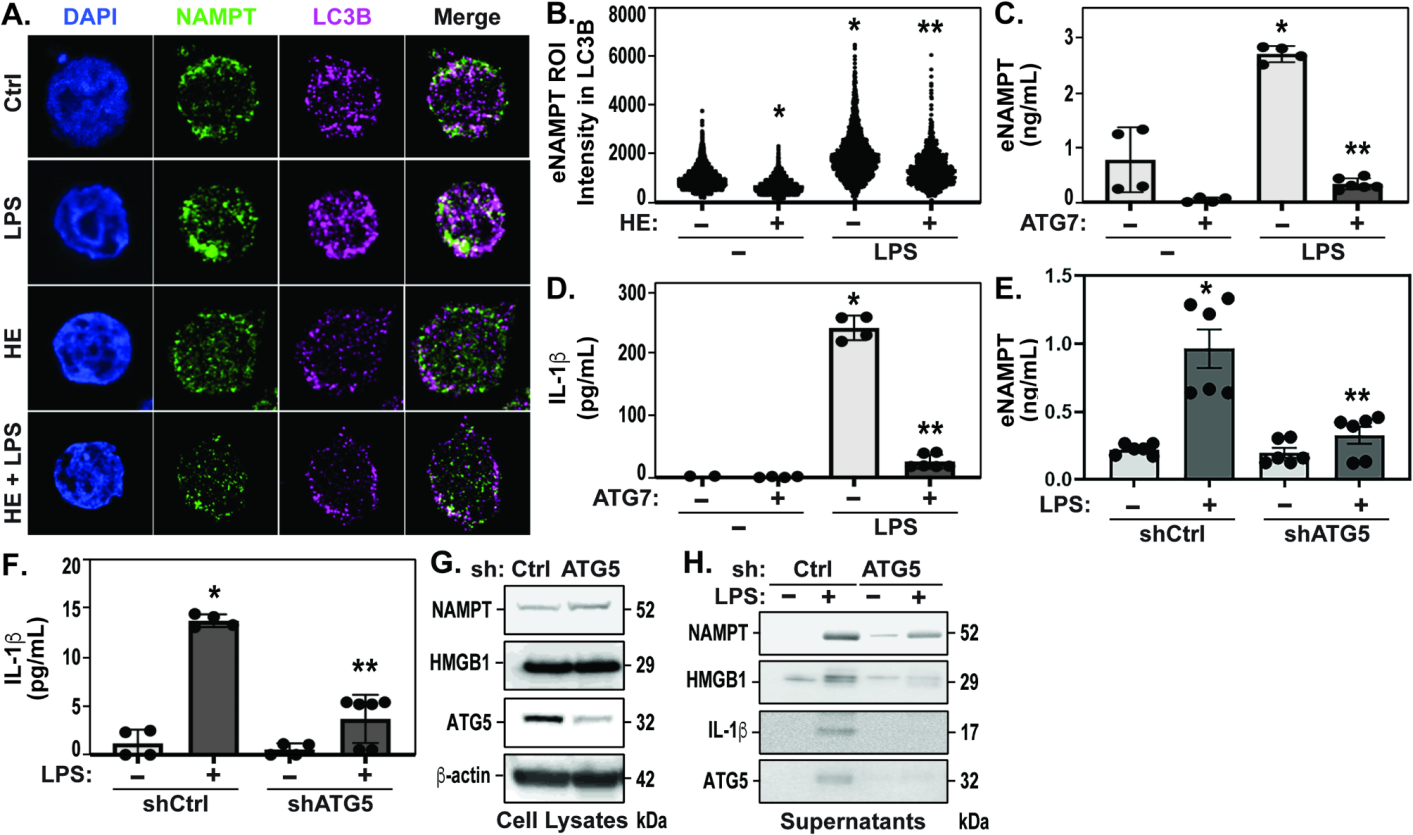
Ubiquitinated NAMPT is recruited to LC3B-containing autophagosomes facilitating autophagy-mediated secretion. (**A/B**) Translocation of NAMPT in LC3B-positive autophagosomes is depicted by super resolution representative microscopy of immunofluorescence with NAMPT and LC3B antibodies. THP-1 WT cells treated with PMA overnight attached to coverslips were treated for 1 h with Heclin (50 µM) followed by LPS 4 h. The coverslips were mounted onto slides for super resolution imaging at 100X. Region of interest (ROI) co-localization of NAMPT fluorescence intensity within LC3B structures was performed utilizing ImageJ Analyze Particles tool. (**C/D**) WT THP-1 cells (~ 1 × 10^6^) were pre-treated with ATG7-IN-1 (40 µM) for 1 h, followed by 4 h of LPS (1 µg/mL). Cell supernatants were collected for MSD assay for detection of eNAMPT and IL-1β secretion. (**E/F**) THP-1 shRNA control (shCtrl) and shRNA ATG5 (shATG5) expressing cells were exposed to LPS (1 µg/mL, 4 h). Cell supernatants were run on ELISA MSD assay for detection of secreted eNAMPT and IL-1β. (**G**) ATG5 knockdown was evaluated by immunoblotting against ATG5, NAMPT, and HMGB1 in WCL. (**H**) Supernatants of shCtrl- and shATG5-transduced THP-1 cells were immunoblotted against eNAMPT, HMGB1, cleaved IL-1β (active), and ATG5 after LPS challenge (4 h). Statistical analysis t-test. Values are expressed as mean Standard error mean (C&D, *p < 0.05 vs. Ctrl, **p < 0.05 vs. LPS; E&F, *p < 0.05 vs. siCtrl, **p < 0.05 vs. LPS)

Due to NEDD4 inhibition reducing NAMPT ubiquitination and secretion, we investigated whether ubiquitinated NAMPT is a cargo protein that undergoes secretion via autophagy. We performed immunofluorescence assays for NAMPT and LC3B in THP-1 cells using super-resolution microscopy to determine if NAMPT localizes to LC3B-positive autophagosomes. Indeed, we observed a significant increase in LC3B fluorescence intensity following LPS challenge in comparison to control cells, as well as an increase in co-localization between NAMPT and LC3B (Fig. 4A/B) which was significantly reduced following Heclin pre-treatment to inhibit NEDD4-mediated NAMPT ubiquitination. Pearson’s and Manders coefficient analyses using the ImageJ JACoP plugin [50] confirmed an increased correlation (R-value) between NAMPT and LC3B after LPS stimulation compared to controls, with a notable decrease in THP-1 cells pretreated with Heclin (Table 1). Additionally, visualization of pretreated THP-1 cells with Heclin allowed for diminished detectable NAMPT fluorescent intensity ROIs to LC3B puncta, consistent with previous NEDD4 pharmacological inhibition studies resulting in eNAMPT attenuation following LPS stimulation (Fig. 4B). These results suggest NEDD4-catalyzed ubiquitinated NAMPT is recruited to LC3B-positive autophagosomes for extracellular export.

**Table 1:** NAMPT co-localization with LC3B

Mander’s and Pearson’s coefficients for NAMPT co-localization with LC3B			
	Mander’s Coefficient		Pearson’s Coefficient
	LC3B/NAMPT	NAMPT/LC3B	
**THP-1 WT** **(control)**	**0.296**	**0.239**	*r* = 0.706
**THP-1 WT** **(LPS)**	**0.262**	**0.397**	*r* = 0.755
**THP-1 WT** **(Heclin)**	**0.709**	**0.369**	*r* = 0.568
**THP-1 WT** **(Heclin + LPS)**	**0.751**	**0.545**	*r* = 0.662

**Fig. 5:**
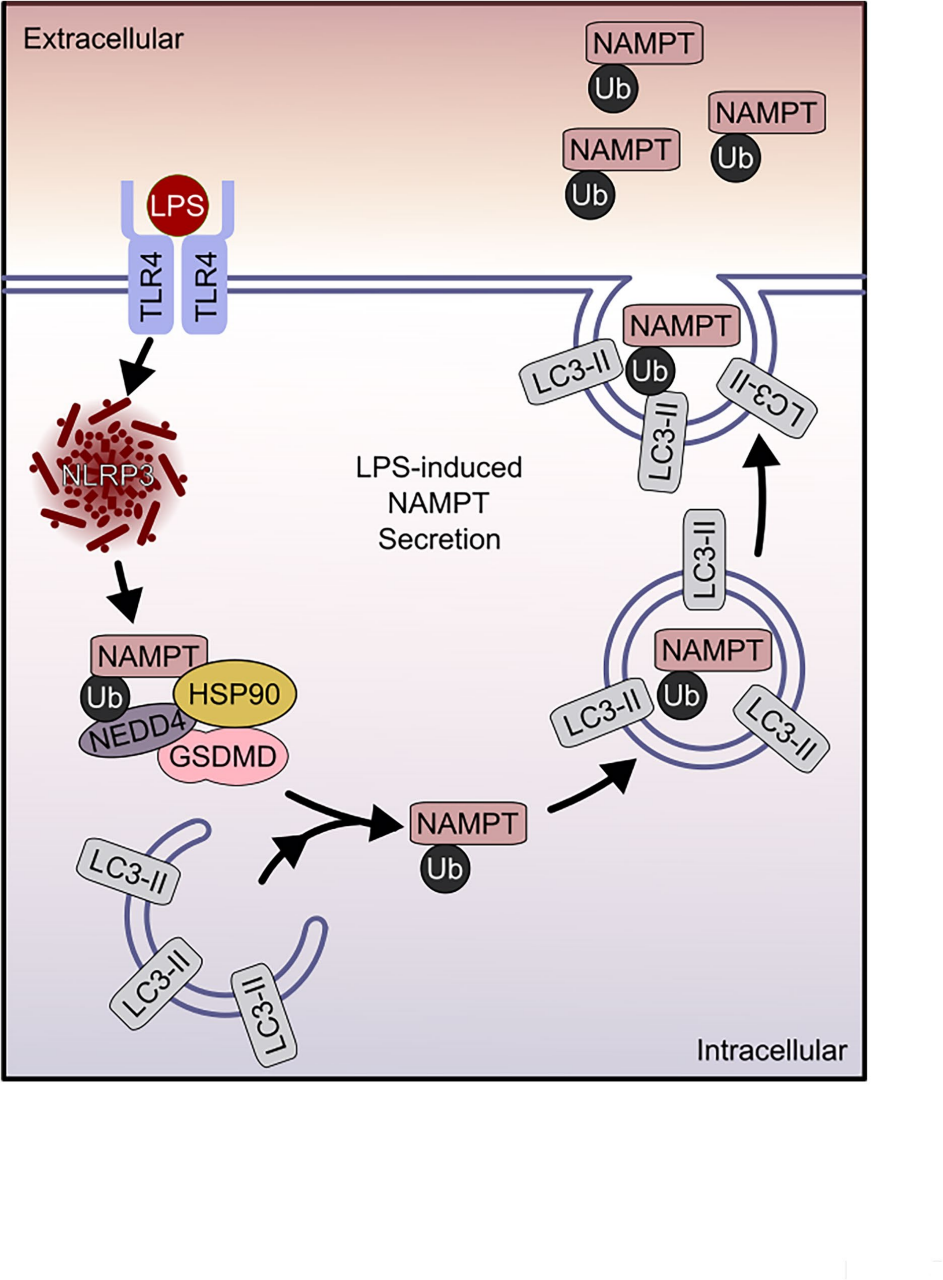
Schematic illustration of non-pyroptotic LPS-induced eNAMPT secretion. Non-pyroptotic secretion of eNAMPT is triggered by LPS binding to TLR4, leading to NLRP3 inflammasome activation. Unlike Nigericin-induced pyroptosis where eNAMPT release requires GSDMD cleavage, pore formation and membrane rupture, LPS-induced eNAMPT secretion requires full-length GSDMD as a key component of a NAMPT ubiquitination complex, involving the NEDD4 E3 ligase and the Hsp90 chaperone protein, and is essential for LPS-induced NAMPT ubiquitination. The ubiquitinated NAMPT is then sequestered within LC3B-positive secretory, non-degradative autophagosomes which are released into the extracellular space with eNAMPT secretion. The secreted eNAMPT acts as a critical TLR4-interacting DAMP, promoting innate immune signaling that regulates inflammatory, fibrotic, and neoplastic processes

This involvement of autophagy machinery in the trafficking of NAMPT as a cargo substrate prompted investigation of non-lysosomal or unconventional autophagy pathways that bypass lysosomal recruitment, particularly those involving LC3B lipidation. IL-1β exemplifies type III UPS mechanisms linked to autophagy involving the secretion of cytoplasmic proteins whose export involves intracellular vesicles as transport intermediates [31, 51], executed by the E3 ligase complex ATG12-ATG5-ATG16L1 [52]. Employing pharmacological inhibition of LC3B lipidation with the ATG7 E1 enzyme inhibitor, ATG7-IN-1 [53, 54], we observed LPS-mediated eNAMPT and IL-1β secretion to be significantly reduced in ATG7-IN-1 pretreated THP-1 cells (Fig. 4C/D) indicating that LC3B lipidation processes are involved in the cargo selection of NAMPT to LC3B-positive autophagosomes. We next silenced the expression of ATG5 (via shRNA lentiviral transduction of ATG5), an E3 ligase autophagy component essential for LC3B lipidation, and observed ATG5 silencing to reduce eNAMPT, IL-1β (Fig. 4E/F), and HMGB1 (Fig. 4G) secretion following LPS stimulation. Expression levels of iNAMPT and HMGB1 in cell lysates were not affected by ATG5 silencing thus excluding ATG5 regulation of iNAMPT and HMGB1 degradation (Fig. 4G), results confirmed by analysis of supernatants by Western blotting (Fig. 4H). Together, these data indicate secretory autophagy as the primary pathway for eNAMPT secretion (Fig. 5).

## Discussion

A variety of tissues/cells, including adipocytes, immune effector cells, endothelial cells, cardiomyocytes, neurons, and various cancer cells, have been noted to increase extracellular secretion or release of the DAMP and TLR4 ligand, eNAMPT. As a result, eNAMPT has emerged as a critical influence to the severity of multiple inflammatory, fibrotic, and neoplastic disorders either via direct induction of innate immune responses or by secondary amplification of innate immune-mediated inflammatory cascades. Single nucleotide polymorphisms (SNPs) in the *NAMPT* promoter influence *NAMPT* transcription and secretion and are linked to the susceptibility/severity of ARDS [16, 28], pulmonary hypertension [23], coronary artery disease [55], diabetes [56], and multiple cancers [57, 58], yet the exact mechanisms by which eNAMPT/TLR4 autocrine/paracrine signaling directly influences disease severity are incompletely understood. eNAMPT/TLR4 activation and sigaling is known to produce the loss of vascular barrier integrity [41], and to induce cellular phenotypic transitions of endothelium [22–24, 59], smooth muscle cells [22, 60], fibroblasts [2, 61], and monocytes/macrophages [62, 63], events that potentially promote vascular remodeling, organ fibrosis and cancer progression. Thus, interrogating the exact mechanisms involved in eNAMPT secretion/release into either the circulation, the perivascular niche or the tumor microenvironment, may prove fundamental to understanding eNAMPT involvement in human pathobiology.

In this study, we interrogated eNAMPT secretion/release from THP-1 monocytes and identified canonical and non-canonical pathways intimately involved in eNAMPT secretion/release in response to Nigericin and LPS. We showed in THP-1 monocytes that potassium influx elicited by Nigericin to result in canonical NLRP3 inflammasome activation, caspase-1-mediated gasdermin D (GSDMD) cleavage to form plasma membrane pores resulting in the release of eNAMPT in a manner similar to IL-1β release [34]. Under these conditions, caspase-1 cleavage and GSDMD activation lead to the processing and secretion of IL-1β, as well as pyroptotic plasma membrane rupture (PMR), which in turn releases DAMPs such as eNAMPT and HMGB1 [36, 37, 64, 65]. In conventional NLRP3 inflammasome activation triggered by Nigericin challenge, the activation of gasdermin D (GSDMD) is essential for pyroptosis via a process involving the caspase-1-mediated cleavage of GSDMD at aspartate 275 (D275) in humans to produce N- and C-terminal GSDMD fragments (NT-GSDMD) [66]. The N-terminal fragments oligomerize and form transmembrane GSDMD pores that facilitate the release of pro-inflammatory cytokines such as IL-1β and IL-18 [65, 67]. These pores, however, ultimately lead to PMR, and as shown in the current study, the pyroptotic release of eNAMPT. We demonstrated that caspase-1 deficiency blocks N-terminal GSDMD cleavage and eNAMPT release in Nigericin-treated THP-1 cells, confirming that NT-GSDMD generation is caspase-1-dependent and involves GSDMD cleavage at D275 as Nigericin-stimulated cells expressing a D275A GSDMD mutant exhibited caspase-1 activation without evidence of D275A GSDMD cleavage, or NT-GSDMD mediated pore formation. The D275A GSDMD mutation resulted in blockage of eNAMPT and HMGB1 release following Nigericin stimulation.

In contrast to Nigericin, our data indicates that extracellular LPS/TLR4 signaling in THP-1 monocytes activates the NLRP3 inflammasome and eNAMPT secretion in the absence of GSDMD cleavage/oligomerization, pore formation or pyroptosis as the GSDMD D275A mutation failed to alter LPS-induced eNAMPT release, results consistent with the absence of a requirement for GSDMD pore formation and PMR/pyroptosis for LPS-induced DAMP secretion. This finding is further supported by the observation that disulfiram, a known inhibitor of GSDMD pore formation, significantly reduced the release of eNAMPT and HMGB1 in Nigericin-treated cells. Importantly, intact GSDMD is essential to LPS-mediated eNAMPT secretion as GKO cells showed loss of eNAMPT and HMGB1 secretion after LPS stimulation. These findings are consistent with recent studies indicating extracellular LPS to induce IL-1β secretion from human monocytes via NLRP3 inflammasome activation without pyroptosis [43–46] with involvement of secretory autophagosomes [47, 48] and heat shock protein 90 (Hsp90) [49].

To more clearly delineate GSDMD involvement in LPS/TLR4-mediated eNAMPT secretion beyond NLRP3 inflammasome activation, we conducted experiments demonstrating eNAMPT secretion to proceed via regulation of NEDD4 E3 ubiquitin ligase resulting in NAMPT ubiquitination in a GSDMD-dependent manner. We observed a direct GSDMD interaction with NEDD4, both basally and after LPS stimulation, with NEDD4 expression reduced in GKO cells compared to WT cells suggesting that GSDMD plays a role in protein recruitment towards ubiquitin signaling. LPS-mediated NLRP3 inflammasome activation increases NEDD4-dependent NAMPT ubiquitination and eNAMPT secretion is abolished by either NEDD4 depletion or by the NEDD4 HECT domain inhibitor, Heclin.

Examination of the cellular localization of ubiquitinated NAMPT showed co-localization with LC3B-positive autophagosomes after LPS stimulation suggesting that ubiquitinated NAMPT, similar to IL-1β, is an autophagic cargo substrate for secretion. This hypothesis was supported by the effective reductions in eNAMPT secretion by inhibition of ATG7, a key protein required for LC3B lipidation and studies employing ATG5 silencing, also an autophagy protein essential for LC3B lipidation and autophagic vesicle formation. The chaperone protein Hsp90 directly interacts with ATG7 (autophagy-related protein 7) [68], a crucial E1-like activating enzyme in the LC3B lipidation pathway via conjugation of ATG12 to ATG5, a process essential for the formation of the autophagosome [69, 70] and fusion with the plasma membrane. When Hsp90 is inhibited, secretory autophagy is impaired by interrupting Hsp90-GSDMD recruitment of NEDD4- ubiquitinated NAMPT and E1 ATG7 enzymatic initiation of LC3B lipidation, events that reduce LC3B-mediated NAMPT secretion. Our data indicate that Hsp90 plays a critical role in eNAMPT secretion via its participation in the GSDMD–NEDD4-NAMPT complex after LPS stimulation as inhibition of Hsp90 with geldanamycin led to a marked reduction in eNAMPT secretion. Our data conclusively indicate that NEDD4 ubiquitinates NAMPT and is involved in directing the movement of ubiquitinated NAMPT to autophagic vesicles through interactions with leucine-rich regions of LC3B.

Limitations of the current study include the singular focus on eNAMPT secretion/release from THP-1 monocytes. Elucidation of cell-specific NAMPT secretion in macrophages, fibroblasts, and endothelial cells is important and would allow for a more comprehensive targeting strategy of pathological innate immune responses involving elevated levels of circulating eNAMPT. Another limitation of this work is the incomplete determination of the ubiquitination status of eNAMPT secreted into the extracellular space, an important lingering question that would inform how eNAMPT signaling is amplified among different cell types and tissues. It is known that the autophagosome may fuse with other intracellular organelles, such as multivesicular bodies or lysosomes, particularly when the inner membrane of the autophagosome is degraded, to promote IL-1β release by amphisome fusion with the plasma membrane of surrounding cells [71]. Our studies did not assess the potential involvement of the “endosomal sorting complexes required for transport” (ESCRT) system in eNAMPT secretion/release. ESCRT proteins are key regulators of exosome formation and cargo packaging [72, 73] and are required for IL-1β ubiquitination and exosomal release [74]. Our results are compatible, however, with studies in LPS-stimulated mouse intestinal epithelial cells where NLRP3 inflammasome activation led to formation of a complex that includes intact GSDMD, Hsp90, the co-chaperone CDC37, and NEDD4, that facilitates pro-IL-1β polyubiquitination and release through CD63-positive exosomes [49].

In summary, these results provide novel insights into the mechanisms underlying non-pyroptotic eNAMPT secretion and pyroptotic eNAMPT release **(**depicted in Fig. 5), a critical innate immunity effector that directly influences the magnitude and severity of innate immune responses. We observed bifurcation of eNAMPT secretion and release to utilize distinct signaling pathways although each pathway was entirely dependent upon activation of the NLRP3 inflammasome. Pyroptotic release of DAMPs evolves via GSDMD pore formation, and lytic cell death to release eNAMPT and HMGB1 into the extracellular space [75] potentiating inflammatory responses via activation of pathogen recognition receptors, such as TLR4 [76–78]. In contrast, LPS/TLR4-mediated eNAMPT secretion follows a tightly choreographed series of propagating biochemical events without pyroptosis induction but is highly dependent on the involvement of key molecular mediators such as NEDD4, Hsp90, and autophagy-related proteins. We speculate that the elucidation of the key mechanisms driving eNAMPT secretion/release allow for targeting of secretory pathways and identification of novel therapeutic approaches to mitigate eNAMPT/TLR4 signaling and innate immune-mediated enhanced severity of inflammatory, fibrotic and neoplastic diseases, a major global unmet medical need.

## Electronic supplementary material

Below is the link to the electronic supplementary material.

Supplementary Material 1

## Data Availability

No datasets were generated or analysed during the current study.

## Abbreviations

ARDS: Acute respiratory distress syndrome
ASC: Apoptosis-associated speck-like protein containing a caspase recruitment domain
ATG5: Autophagy protein 5
ATG7: Autophagy related 7
Cas-1: Caspase-1
DAMP: Damage associated molecular pattern protein
DSF: Disulfiram
eNAMPT: Extracellular NAMPT
ER: Endoplasmic reticulum
ESCRT: Endosomal sorting complexes required for transport
GA: Geldanamycin
GKO: GSDMD knockout
GSDMD: Gasdermin D
HE: Heclin
HMGB1: High mobility group box 1
Hsp90: Heat shock protein 90
IL-1β: Interleukin-1 beta
iNAMPT: Intracellular NAMPT
KD: Knock down
KO: Knockout
LC3B: Microtubule-associated protein 1 light chain 3
LDH: Lactate dehydrogenase
LPS: Lipopolysaccharide
MSD: Meso scale discovery
NAD: Nicotinamide adenine dinucleotide
NAMPT: Nicotinamide phosphoribosyltransferase
NEDD4: Neural precursor cell expressed developmentally downregulated 4
NKO: NLRP3 knockout
NLRP3: Nucleotide-binding domain (NOD)-like receptor protein 3
NMN: Nicotinamide mononucleotide
NMNAT: Nicotinamide mononucleotide adenylyltransferase
NT-GSDMD: N-terminal GSDMD
PAMPs: Pathogen-associated molecular patterns
PFA: Paraformaldehyde
PMA: Phorbol 12-myristate-13-acetate
PMR: Plasma membrane rupture
PRRs: Pattern recognition receptors
THP-1: Tohoku Hospital Pediatrics-1
TLR4: Toll-like receptor 4
UPS: Unconventional protein secretion
