# Supplementary material for: NEDD4 E3 ligase-catalyzed NAMPT ubiquitination and autophagy activation are essential for pyroptosis-independent NAMPT secretion in human monocytes
Source: Cell Commun Signal. 2025 Mar 30;23:157. doi: 10.1186/s12964-025-02164-5 (PMC11956250; doi:10.1186/s12964-025-02164-5)
Supplement: Supplementary file 1 — Supplementary Material 1 [file 12964_2025_2164_MOESM1_ESM.pdf]

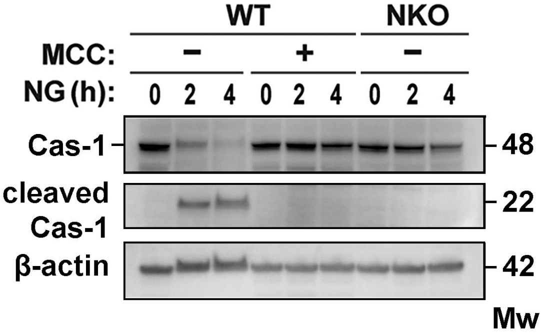
WT
NKO
MCC: − + −
NG (h): 0 2 4 0 2 4 0 2 4
Cas-1
cleaved Cas-1
β-actin
48
22
42
Mw

Supplemental Figure S1. Nigericin induces caspase-1 cleavage via NLRP3. Immunoblotting detection of caspase-1 cleavage. MCC-950-pretreated (20 μM, 1 hr) and untreated WT and NKO THP-1 cells were stimulated with Nigericin (10 μM) for 2 and 4 hours.

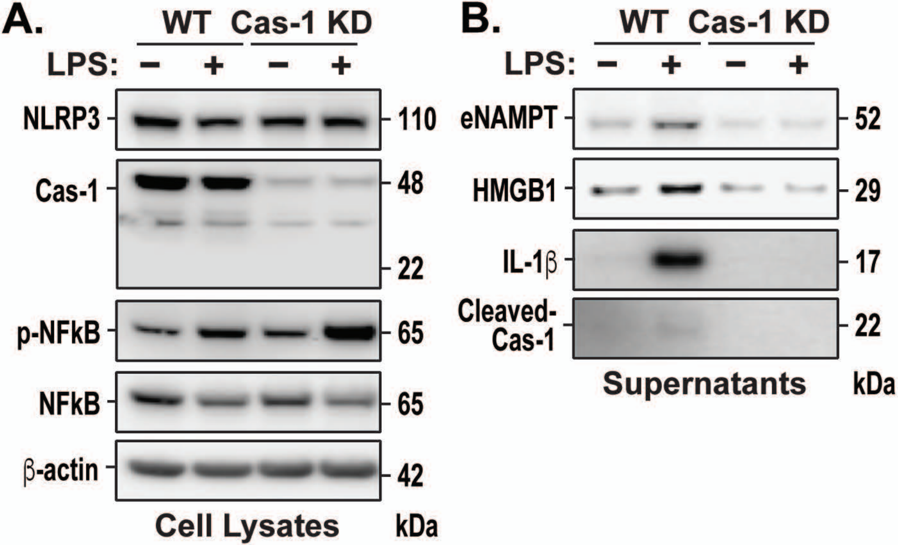
A.
WT
Cas-1 KD
LPS: − + − +
NLRP3
110
Cas-1
48
22
p-NFkB
65
NFkB
65
β-actin
42
Cell Lysates
kDa
B.
WT
Cas-1 KD
LPS: − + − +
eNAMPT
52
HMGB1
29
IL-1β
17
Cleaved-Cas-1
22
Supernatants
kDa

Supplemental Figure S2. Caspase-1 deficiency reduces LPS-induced eNAMPT secretion. (**A**) THP-1 wild type (WT) and caspase-1 deficient (Cas-1 KD) cells were treated for 4 hr with 1 μg/mL LPS. Caspase-1 deficiency in Cas-1 KD cells was confirmed by immunoblotting total cell lysates. Both WT and Cas-1 KD cells showed increased phosphorylation of NF-κB after LPS exposure. Immunoblots for total NF-κB, NLRP3, and β-actin are shown. (**B**) Supernatants from THP-1 WT and Cas-1 KD cells treated with LPS were immunoblotted for eNAMPT, HMGB1, cleaved IL-1β (active), and cleaved caspase-1. LPS-induced secretion of eNAMPT, HMGB1, and IL-1β was markedly reduced in Cas-1 KD cells, compared to WT cells.

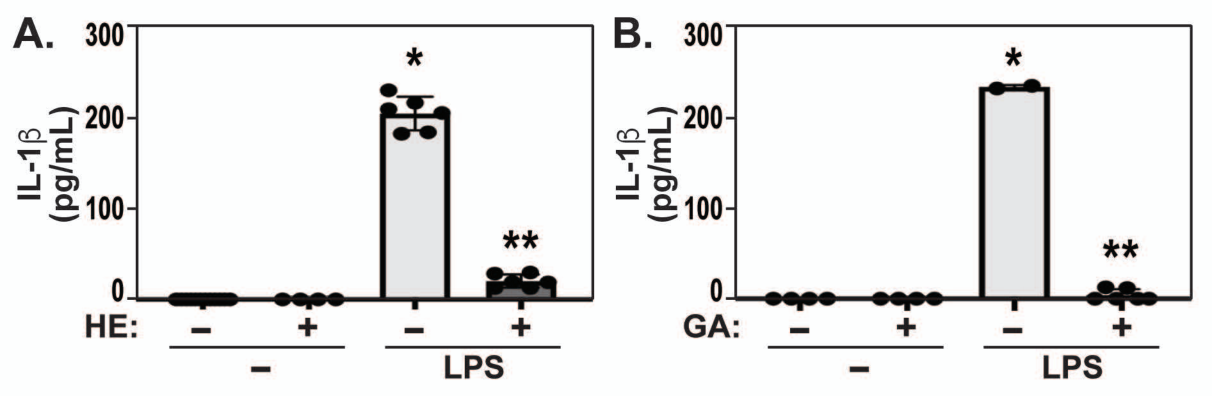

A.
300
200
100
0
IL-1β
(pg/mL)
*
**
HE:
–
+
–
+
–
LPS
B.
300
200
100
0
IL-1β
(pg/mL)
*
**
GA:
–
+
–
+
–
LPS

Supplemental Figure S3. LPS-induced IL-1β secretion is inhibited by Heclin and Geldanamycin treatment. (**A/B**) THP-1 WT cells were pretreated for 1 hr with either 50 μM Heclin (HE) or 30 μM Geldanamycin (GA), followed by 4 hr of treatment with 1 μg/mL LPS. IL-1β concentrations in the supernatants were measured using MSD, and results are shown as the mean ± SEM of four independent experiments.
